# Total wrist arthroplasty revisited: survival, function, and patient-reported outcomes of BIAX and universal II prostheses over 15 years

**DOI:** 10.1007/s00402-025-06005-5

**Published:** 2025-07-30

**Authors:** Leonhard Mandl, Alfred Gruber, Raymund. E. Horch, Resit Demir

**Affiliations:** 1 Universitätsklinikum Erlangen, Erlangen, Germany; 2 Medical Centre Demir, Nuernberg Germany, Germany; 3 Medical Centre Demir, Nuernberg Germany, Germany

**Keywords:** Total Wrist Arthroplasty, BIAX, Universal II

## Abstract

**Background:**

In the management of degenerative osteoarthritis and rheumatoid arthritis of the wrist, surgical intervention remains an important treatment option when conservative approaches such as medication, physiotherapy, and assistive devices prove insufficient. Despite decades of development, no single wrist prosthesis has yet emerged as the definitive standard in total wrist arthroplasty. To address this gap, the German Society for Hand Surgery established the Total Endoprosthesis (TEP) Register in 2004 to systematically collect and evaluate long-term clinical outcomes. The present prospective single-centre study analyses and presents the long-term results of patients enrolled in the TEP register, offering valuable insights into the performance and durability of wrist prostheses in a real-world clinical setting.

**Patients/materials/methods:**

Between 2000 and 2011, a total of 29 total endoprostheses were implanted in 25 patients in a single centre study due to severe degenerative changes in the wrist. Of these, 22 had rheumatoid arthritis and 3 degenerative osteoarthritis. The prosthesis models BIAX (*N* = 13) and Universal II (*N* = 16) were implanted. The present study describes the mean outcome after a mean observation period of 13.8 (1.7–21.4) years for the BIAX prosthesis and 10.5 years (0.2–16.4) for the Universal II prosthesis.

**Results:**

The probability of survival after 15 years is 60% for the BIAX prosthesis and 41% for the Universal II prosthesis. After 21 years, results are only available for the BIAX prosthesis with a value of 60%. The BIAX prosthesis showed a lower complication rate. Subjectively, 15 years postoperatively, the BIAX prosthesis showed a lower mean value in the QuickDASH score (BIAX 33.8%, Universal II 50.3%), lower results in the visual analogue pain scale under stress (BIAX: 1 point; Universal II: 3 points) with higher patient satisfaction (BIAX: 86%; Universal II: 78%).

**Conclusion:**

In conclusion, our observations show that the BIAX prosthesis is superior to the Universal II prosthesis 15 years after surgery in terms of survival probability, complication rate and better results in satisfaction, the visual analogue pain scale and the QuickDASH score.

## Introduction

In patients with osteoarthritis or rheumatoid arthritis, a primary challenge in wrist joint reconstruction is achieving effective pain relief while preserving as much range of motion as possible through total wrist arthroplasty (TWA) also known as total endoprosthesis (TEP). Although arthrodesis remains the most reliable method for long-term pain control, it significantly compromises joint mobility and is therefore often unsatisfactory for many patients. In contrast, TWA allows for preserved joint motion but is associated with higher rates of prosthetic loosening as range of motion increases, and typically provides less substantial pain relief compared to arthrodesis.

To improve clinical outcomes and enable comparative evaluation, a wrist TWA registry was established to facilitate centralized documentation of long-term results, support early detection of prosthesis failure, and allow assessment of different wrist prosthesis systems. However, comprehensive long-term data on wrist prostheses remain scarce. This study aims to contribute valuable findings to the registry by analyzing the long-term outcomes of wrist TWA in a predominantly arthritic patient cohort.

### Patients

Between 2000 and 2011, one surgeon implanted a total of 29 total endoprostheses (13 BIAX and 16 Universal II) in 25 patients in a single centre study due to severe degenerative changes in the wrist. Of these, four patients were each implanted with two TEPs, as they either received one prosthesis on both wrists or two prostheses on one wrist as a result of a revision. The gender distribution shows 7 male and 18 female patients, which corresponds to a male to female ratio of 1:2.6. The average age at the time of surgery was 51 (29–79) years. Of these, 22 had rheumatoid arthritis and 3 had degenerative osteoarthritis, with the latter only affecting male patients. The average BMI of the operated patients was 24 kg/m² (17–37). 4 of the 29 prostheses were cemented: 1 BIAX and 3 Universal II prostheses. 2 of the Universal II prostheses replaced the BIAX prosthesis that had been replaced following a revision. Most of the prostheses (59%) were implanted in the right wrist. The average duration of surgery was 125 (85–185) minutes.

## Materials and methods

The study involved the implantation of two prosthesis models: the BIAX and the Universal II. All patients provided informed consent for both the surgical procedure and subsequent follow-up examinations, which were conducted in accordance with the total endoprosthesis (TEP) registry of the German Society for Hand Surgery. Data were collected prospectively during routine clinical assessments, documenting both preoperative and postoperative parameters.

Functional impairment was assessed using the standardized Disabilities of the Arm, Shoulder, and Hand (DASH) questionnaire (0% = no impairment, 100% = maximum impairment), while subjective pain levels were evaluated using a visual analogue scale (VAS) ranging from 0 (no pain) to 10 (maximum pain). Additionally, range of motion (ROM) measurements were recorded. At the final follow-up, patient satisfaction was assessed using a 5-point Likert scale (1 = very satisfied, 5 = not at all satisfied), and patients were asked whether they would recommend the procedure (yes/no). The degree of wrist load in daily life was also surveyed.

Outcomes associated with the BIAX prosthesis were compared with those of the Universal II model. Due to the limited sample size, no control group was included. The dataset was supplemented with information from the latest follow-up. Statistical analyses were performed using IBM SPSS Statistics, version 29.0.

The endpoint of follow-up was defined as prosthesis revision or explantation followed by wrist arthrodesis. In cases where patients were not available for the final follow-up, data from their most recent clinical examination were used, and the follow-up duration was adjusted accordingly. Patients who underwent explantation with subsequent arthrodesis were considered study dropouts from the time of fusion. However, if a new prosthesis was implanted following explantation, the patient remained in the study, with the newly implanted device evaluated independently regarding complications and prosthesis survival.

## Results

25 endoprothesis were implanted in 7 men and 18 women with a mean operation time of 125 (85–185) minutes and a mean age of 53 (30–84) years. Of these, 22 patients had rheumatoid arthritis and 3 patients had osteoarthritis (Table 1).

**Table 1:** Baseline of the 25 patients

Baseline of the 25 patients	
Variable	*n* = 25
Gender	
Male	7
Female	18
OP age in years	
Median	51
Range	29–79
Rheumatoid arthritis	22
Osteoarthritis	3
Duration of operation in min.	
Mean value	125
Range	85–185

Of the 29 prostheses, 17 were implanted on the right and 12 on the left, of which 13 were BIAX and 16 Universal II prostheses. 4 prostheses were cemented and 25 were uncemented. At the time of follow up the BMI of the patients was 24 (17–37) (Table 2).

**Table 2:** Baseline of the 29 implanted prostheses

Baseline of the 29 implanted prosthesis	
Variable	*n* = 29
Operated side	
Right	17
Left	12
BIAX prosthesis	13
Universal II prosthesis	16
Cemented	4
Non cemented	25
BMI	
Mean value	24
Range	17–37

The mean follow-up for the BIAX prosthesis is 13.8 (0.7–21.4) years and 9.8 (0.2–16.4) years for the Universal II prosthesis. The longer observation period of the BIAX prosthesis is due to the earlier implantation. On average, patients had the BIAX prosthesis implanted for 13.7 (1.7–21.4) years, compared to 10.4 (4.8–16.4) years for the Universal II prosthesis. In comparison, the probability of survival after 5 years is 12% lower for the BIAX prosthesis (80%) than for the Universal II prosthesis (92%). After 10 years, this probability of survival is 8% higher for the BIAX prosthesis (70%) than for the Universal II prosthesis (62%). The greatest difference is found in the group ≥ 15 years: Here, the survival probability for the BIAX prosthesis is 19% higher at 60% than for the Universal II prosthesis at 41%. For a survival time of 17 years and longer, the results could only be calculated for the BIAX prosthesis. At 21 years, the probability of survival is 60% (Tables 3 and 4).

**Table 3:** Probability of survival in percent: BIAX prosthesis

Probability of survival in percent: BIAX prosthesis	
Variable in percent	years
80	5
70	10
60	15
60	21

**Table 4:** Probability of survival in percent: universal II prosthesis

Probability of survival in percent: Universal II prosthesis	
Variable in percent	years
92	5
62	10
41	15

**Fig. 1:**
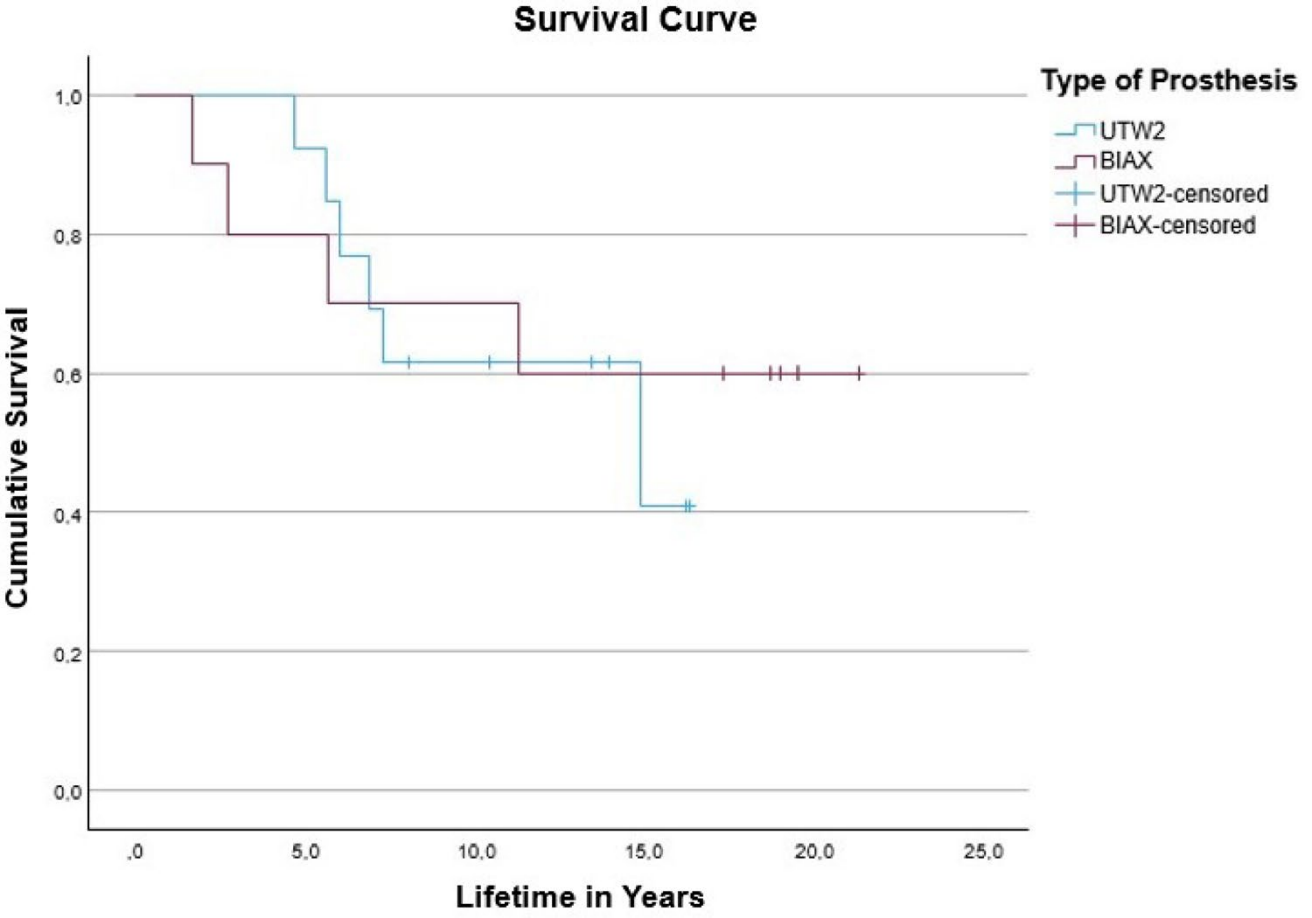
Survival time of the BIAX and universal II prosthesis

In the overall comparison, the long-rank test of both survival curves results in a significance of 0.73, meaning that the difference between the two survival curves is not significant. The following graph shows the cumulative survival relative to the service life in years (Fig. 1).

The results of the QuickDASH score, the visual analogue pain scale and the range of motion are summarized in Table 5.

**Table 5:** Overview of range of motion, quickdash and pain scale

Overview of range of motion, QuickDASH and pain scale				
	BIAX Prosthesis		Universal II Prosthesis	
	N	Unit	N	Unit
**Range of motion**				
Pronation/Supination	6	82,6°/75°	6	80°/75,6°
Flexion/Extension	6	46,3°/42,6°	6	38,6°/38,6°
Radial/Ulna Abduction	6	13,3°/27,3°	6	13,3°/25,3°
**QuickDASH**				
Preoperative	6	33,8	11	50,3
≥ 15 ≤ 22 Years	6	21,3	2	38,9
**Pain scale**				
≥ 15 ≤ 22 Years after implantation without load	4	1	1	0
≥15 ≤ 22 Years after implantation under load	4	1	1	3

All patients, both those in the BIAX group and the Universal II group, benefited from the operation in terms of DASH score. Contrary to the assumption, there is no progressive increase in the QuickDASH score with a longer service life due to the increasing wear of the prosthesis. On average, the score in the last follow-up group ≥ 15 years ≤ 22 years was reduced by 12.5–21.3% for the BIAX prosthesis and by 11.4–38.9% for the Universal II prosthesis compared to the preoperative baseline value. The BIAX prosthesis achieved lower values with an overall longer survival life.

The results of the visual analogue pain scale regarding pain assessment show that the pain level under load is lower with the BIAX prosthesis from the fifth postoperative year onwards. This can also be seen in the group ≤ 22 years: the score under load is 1 for the BIAX prosthesis and 3 points for the Universal II prosthesis. Without loading, the Universal II prosthesis showed lower values. It is one point. The difference is not significant (Mann-Whitney U-test) in the group ≤ 22 years both without load with a *p* = 1 (SD 1) and with load *p* = 0.4 (SD 1.3).

A similar range of motion was observed for both prosthesis models. With the BIAX prosthesis, the range of motion in pronation/supination was 82.6° (SD 4.6)/75° (SD 8.5), in flexion/extension 46.3° (SD 4)/42.6° (SD 8.7), in radial/ulnar abduction 13.3° (SD 0.6)/27.3° (SD 4). The results of the Universal II prosthesis were 80° (SD 9.1)/75.6° (SD 1.2) in pronation/supination, 38.6° (SD 28.5)/38.6° (SD 1.5) in flexion/extension and 13.3° (SD 2.1)/25.3° (SD 4) in radial/ulnar abduction. The BIAX prosthesis shows a slightly better range of motion in the flexion/extension range. The differences are not significant.

The following table shows the complications of both prostheses (Table 6).

**Table 6:** Complications of both prostheses

Complications of both prostheses				
	BIAX prosthesis *N* = 13		Universal II prosthesis *N* = 16	
	N	%	N	%
Luxation	5	39	3	19
Prosthesis Failure	2	15	2	13
Dislocation	4	31	5	31
Synovial arthritis	2	15	7	44
Pain	3	23	6	38

### Comparative analysis of complications and outcomes between BIAX and universal II prostheses

An analysis of complication profiles revealed distinct patterns for the two prosthesis models. For the BIAX prosthesis, the most frequent complication was dislocation, observed in 5 cases (39%), followed by pain in 3 cases (23%) and synovial arthritis in 2 cases (15%). In contrast, the Universal II prosthesis was most commonly associated with synovial arthritis, affecting 7 cases (44%), followed by pain in 6 cases (38%) and dislocation in 5 cases (31%) (see Table 5).

When recalculating dislocation rates based solely on the cases that underwent radiological follow-up—9 for BIAX and 8 for Universal II—the prevalence increased markedly: 63% for the Universal II prosthesis (5 of 8 cases) and 44% for the BIAX prosthesis (4 of 9 cases). This adjusted comparison emphasizes a higher dislocation tendency in the Universal II group.

The greatest disparity between both prostheses was observed in the incidence of synovial arthritis, with a 29% higher rate in the Universal II group (44%) compared to the BIAX group (15%).

Prosthesis failure—defined as the need for revision or explantation—was relatively low and comparable in both groups: 2 cases (15%) for BIAX and 2 cases (13%) for Universal II. While the overall complication-free rate was marginally higher for the Universal II prosthesis (19%) than for BIAX (15%), the Universal II model exhibited a greater number of complications occurring with frequencies above 30%.

Detailed analysis of dislocations indicated that the distal component was primarily affected: in 80% of BIAX and 100% of Universal II dislocations. Furthermore, dislocation events were time-dependent—70% occurred after more than 5 years of implantation. During this timeframe, dislocations were noted in 60% of BIAX and 80% of Universal II cases. Dislocation was also the leading cause for explantation in both prosthesis types, accounting for 3 BIAX and 2 Universal II explantations among a total of 10 removed implants.

Regarding patient-reported satisfaction, 86% of individuals with the BIAX prosthesis rated their outcome as “very satisfied” or “satisfied”, compared to 78% in the Universal II group—an 8% advantage for BIAX. Notably, none of the patients with the Universal II prosthesis rated their outcome as “not at all satisfied”, while 14% of BIAX recipients reported this lowest level of satisfaction.

In terms of recommendation rate, 75% of BIAX users and 78% of Universal II users would recommend the surgery to others—a 3% advantage for Universal II. Reasons cited for non-recommendation included poor long-term results (e.g., explantation), aesthetic concerns, postoperative inflammation, and insufficient strength recovery.

### Representative case examples

#### Case 1: BIAX prosthesis after explantation of a partial arthrodesis– lifetime 18,8 years

**Fig. 2:**
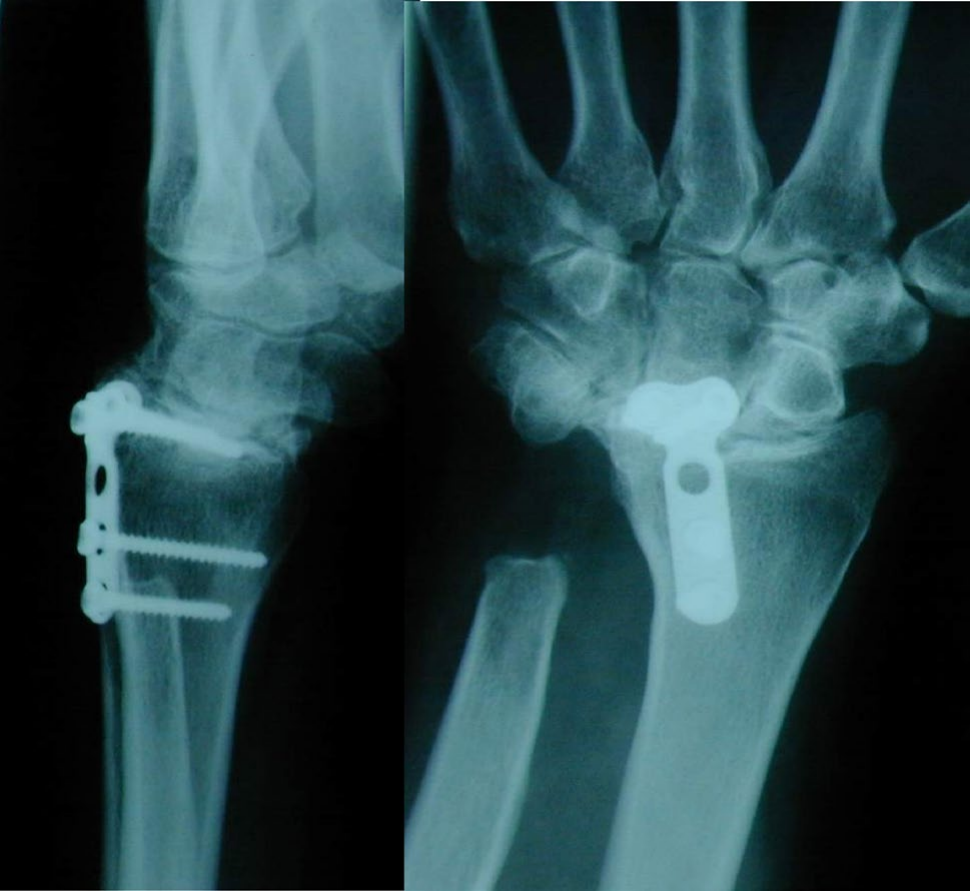
Case 1 - Left preoperative X-ray in lateral and a.p. view

The initial situation is illustrated in Fig. 2, which shows the X-ray of a 45-year-old female patient with rheumatoid arthritis and carpal instability. The patient initially underwent partial arthrodesis with Shapiro staples until 1994, followed by a radiolunate partial arthrodesis until 2002. Both implants were subsequently removed due to persistent carpal instability.

**Fig. 3:**
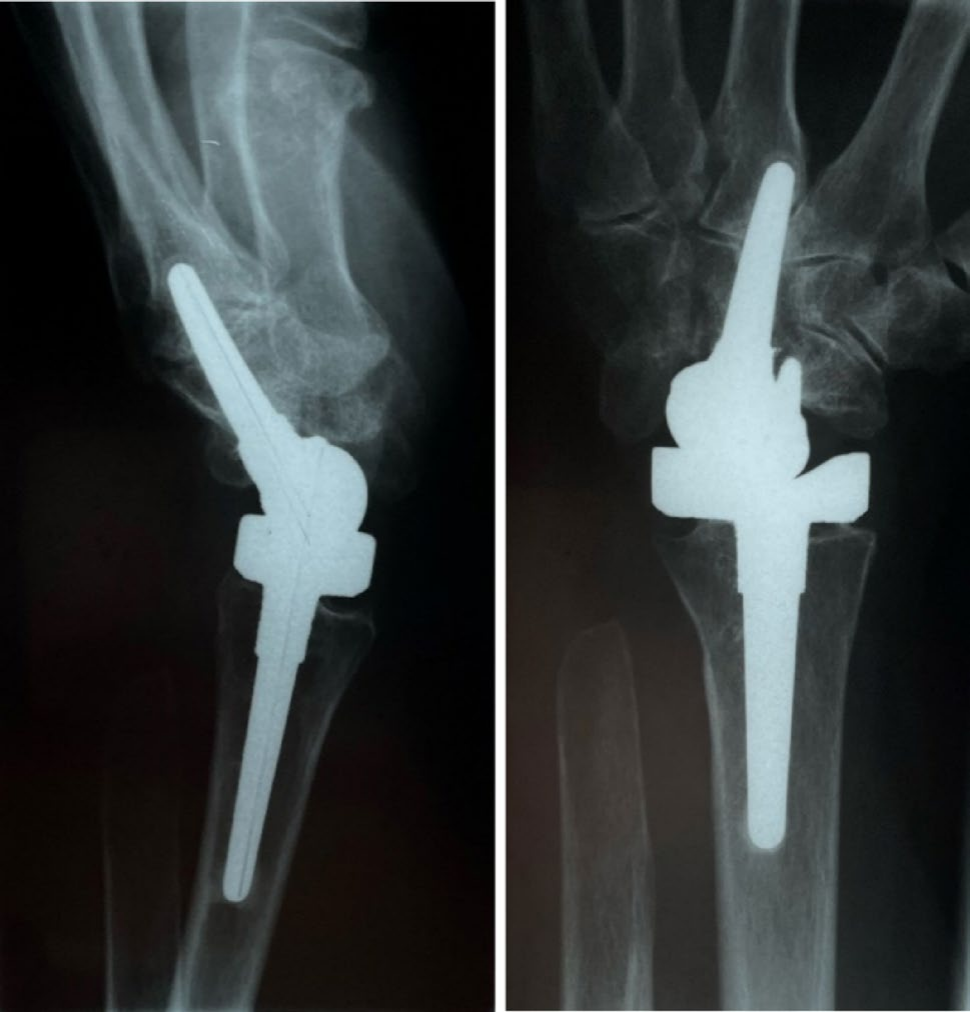
Case 1 - Left postoperative X-ray in lateral and a.p. view

Figure 3 shows the prosthesis well integrated 18.8 years after implantation. In the lateral view, the distal part is tilted dorsally. In the a.p. image, the dorsal part of the prosthesis is not in the centre of the cup, but has migrated through the ulnar shift. There were no postoperative interventions. The patient reports no pain at rest or under load. Her range of motion is 85°/85° in supination/pronation, 50°/55° in extension/flexion and 20°/30° in radial/ulnar abduction. The patient states that she puts up to 30 kg of weight on the wrist in everyday life. Clinically, the hand is characterized by a deviation of the long fingers. There is no swelling of the wrist.

#### Case 2: BIAX-prothesis– lifetime 21,4 years

**Fig. 4:**
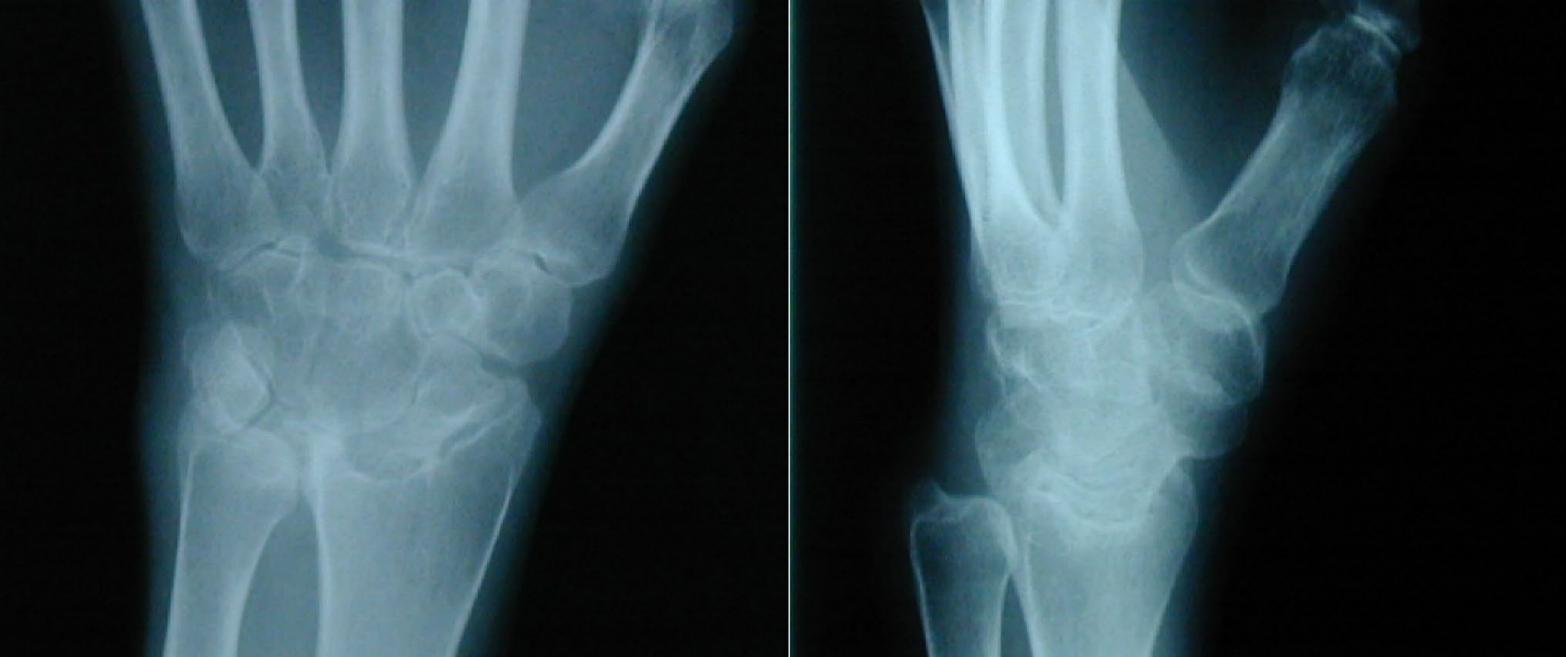
Case 2 - Left preoperative X-ray in lateral and a.p. view

The initial situation (Fig. 4) is a 29-year-old female patient with rheumatoid arthritis. She presents with advanced rheumatoid arthritis of the wrist. The joint space in the radiocarpal joint is closed, there are signs of sclerosis on the scaphoid and lunate as well as on the distal radius.

**Fig. 5:**
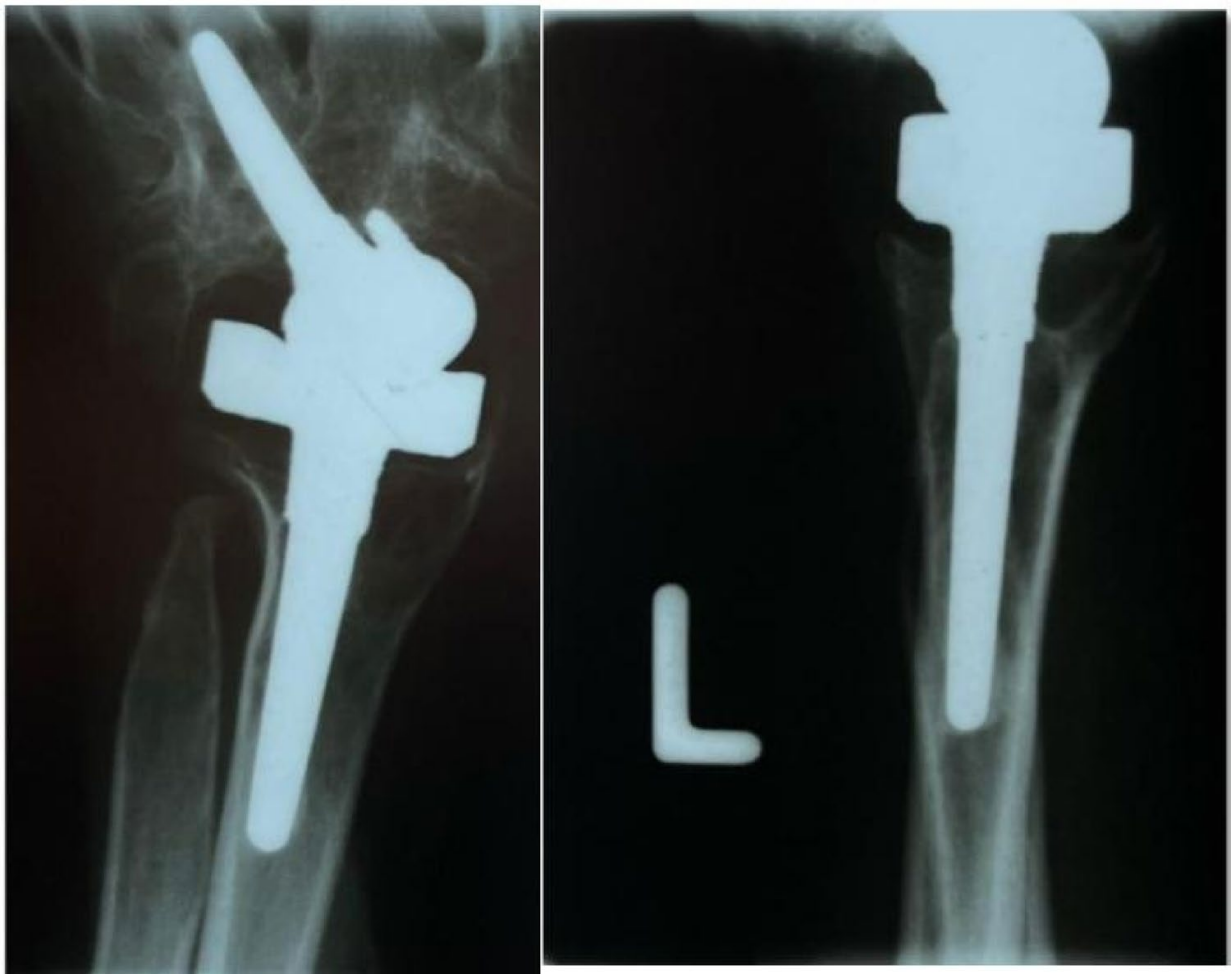
Case 2- Left postoperative X ray in lateral and a.p. view

Figure 5 shows the condition of the left hand 21.4 years after implantation of the BIAX prosthesis. The stem of the proximal and distal part of the prosthesis is in position, there is no osteolysis and no signs of loosening. The service life is the longest in this study. There were no postoperative interventions. The patient has no pain at rest and a value of 2 on the VAS under load. She achieves 90°/60° in pronation/supination, 00°/50° in extension/flexion and 5°/5° in radial/ulnar abduction.

#### Case 3: universal II prosthesis after removal of the BIAX prosthesis– lifetime 16.4 years

**Fig. 6:**
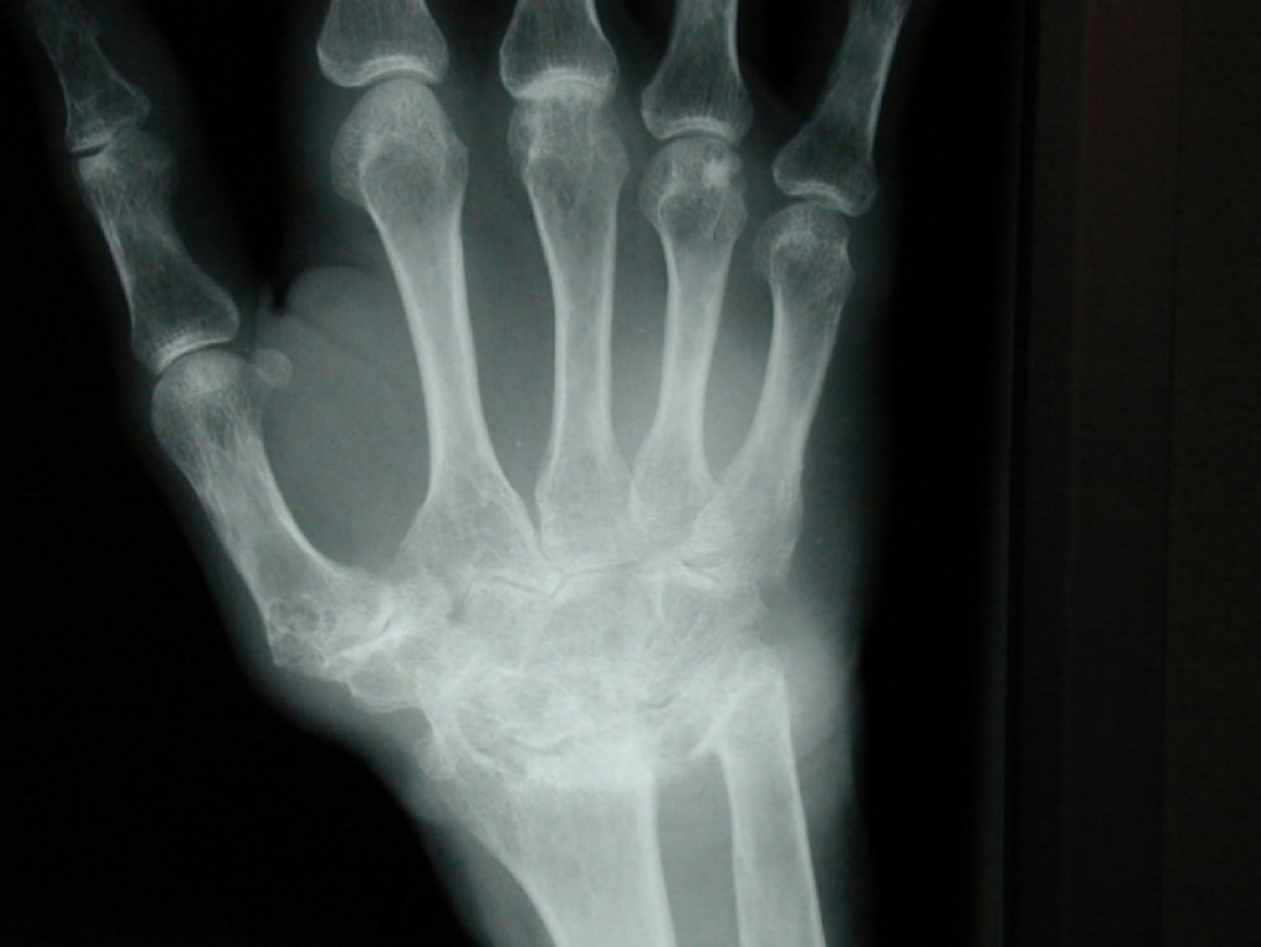
Case 3– Right preoperative X-ray a.p. view

**Fig. 7:**
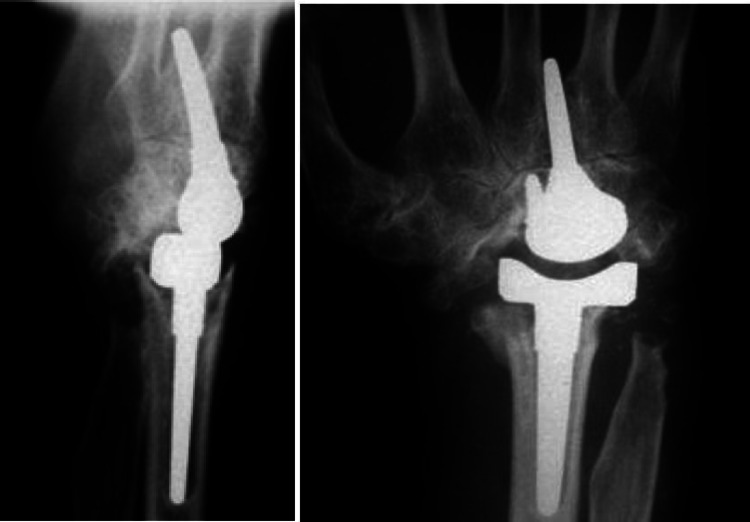
Case 3– Right postoperative X-ray in lateral and a.p. view. Twenty-one months after implantation of a BIAX-prosthesis

The initial situation (Fig. 6) is a rheumatoid 42-year-old patient. He presents with advanced rheumatoid arthritis of the wrist. The joint space in the radiocarpal joint is closed, there are signs of sclerosis on the scaphoid and lunate as well as on the distal radius.

Figure 7 shows twenty-one months after implantation of a BIAX prosthesis. The distal part of the prosthesis has dislocated dorsally from the os metacarpale, the proximal part has sunk into the radius and loosened. The widened intra-articular gap leads to habitual dislocations and pain.

**Fig. 8:**
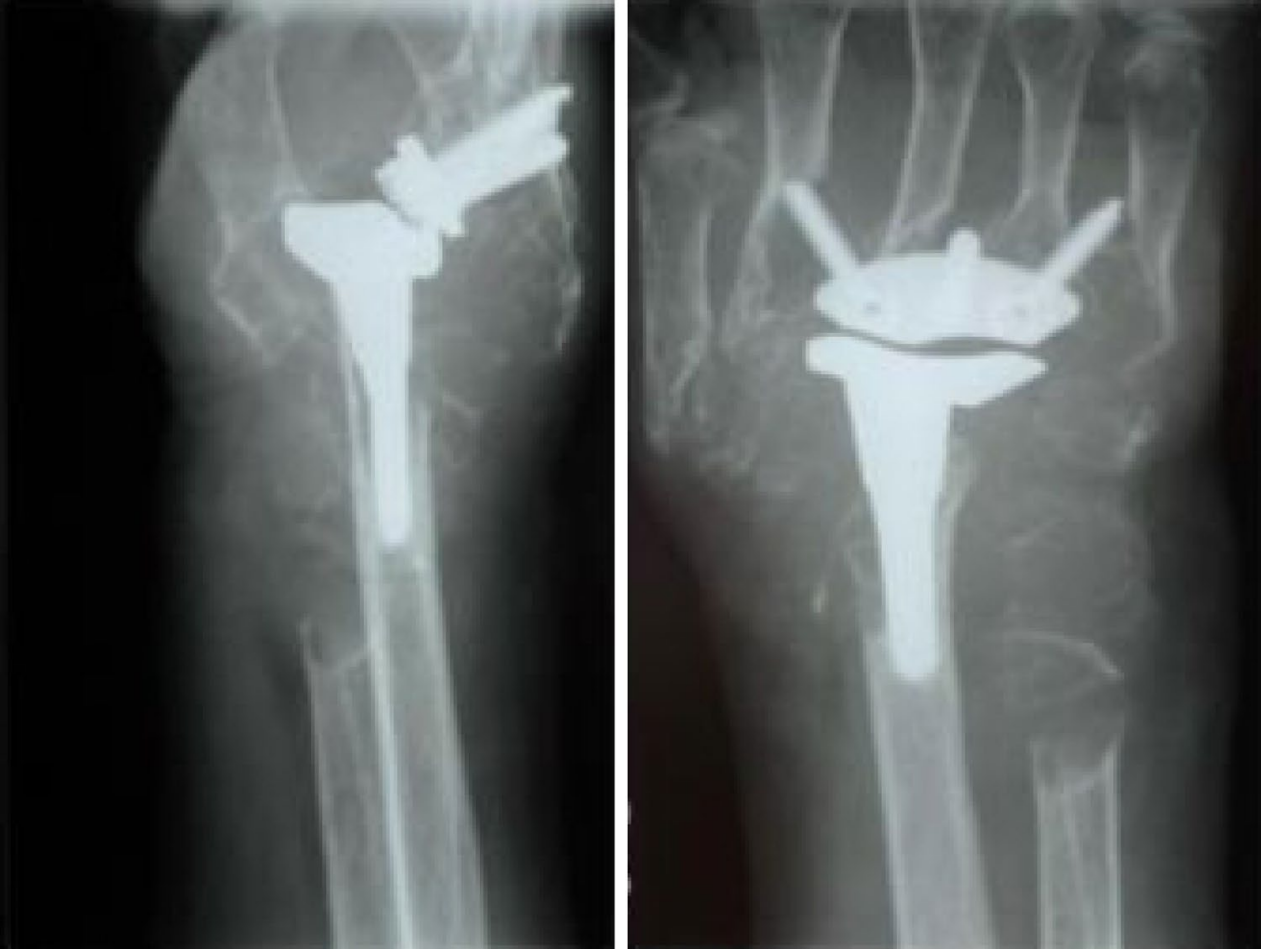
Case 3– Postoperative X-ray of the right wrist (lateral and a.p. view) 16.4 years after revision surgery, replacing the BIAX prosthesis with a Universal II prosthesis

Fig. 8 shows twenty-one months after implantation of a BIAX prosthesis. The distal part of the prosthesis has dislocated dorsally from the os metacarpale, the proximal part has sunk into the radius and loosened. The widened intra-articular gap leads to habitual dislocations and pain

Sixteen point four years after revision surgery with replacement of the BIAX prosthesis by a Universal II prosthesis, the following findings are demonstrated in Fig. 8: The lateral X-ray shows signs of bone resorption and cortical defects at the dorsal edge of the radius around the proximal stem. The carpal component of the prosthesis is dorsally tilted and completely loosened. In the a.p. view, the carpal component is dislocated, and both screws have migrated out of the bone. A dislocated radial screw has caused destruction and partial resorption of the second metacarpal bone. The strut between the third and fourth metacarpal bones is also dislocated. On the Visual Analog Scale (VAS), the patient reports no pain at rest and a pain score of 3 during exertion. The range of motion is 70°/75° in pronation/supination, 50°/70° in extension/flexion, and 15°/30° in radial/ulnar abduction. Clinically, the patient exhibited marked swelling of the wrist. The 62-year-old patient is coping well with his current situation and does not wish to pursue any further treatment.

#### Case 4: BIAX prosthesis following the explantation of a partial carpal arthrodesis - lifetime 19.1 years

**Fig. 9:**
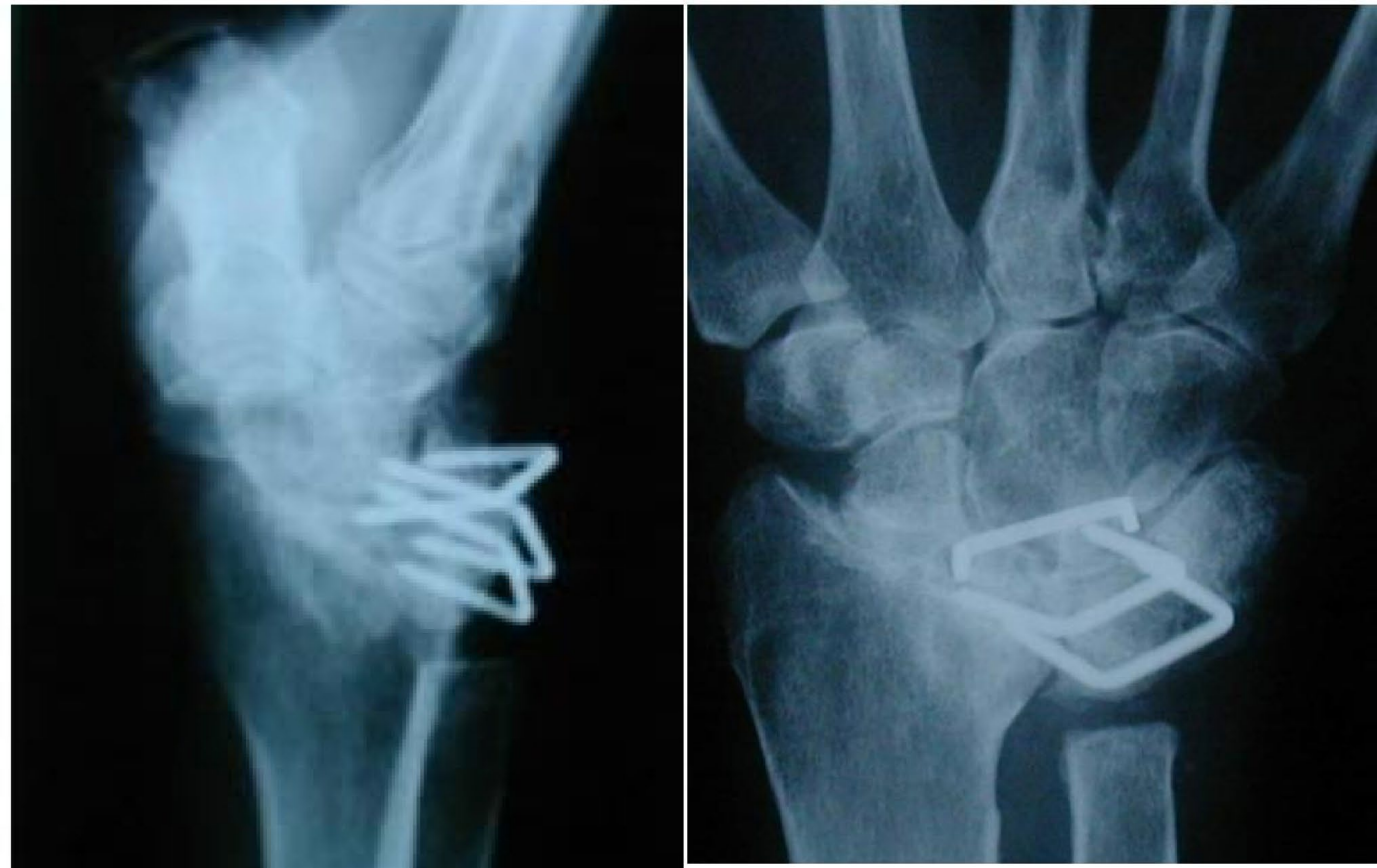
Case 4– Right preoperative X-ray in lateral and a.p. view

The initial situation (Fig. 9) in a 45-year-old female rheumatic patient. The preoperative X-rays of the right wrist (lateral and anteroposterior [a.p.] views) demonstrate a partial wrist arthrodesis using Shapiro staples bridging the carpal bones to the distal radius. There is evidence of implant loosening, with loss of congruity at the fusion site and possible migration or displacement of the staples. The carpus appears malaligned with signs of ulnar translocation. Osteopenic changes and joint space narrowing are noted, consistent with underlying rheumatoid arthritis.

**Fig. 10:**
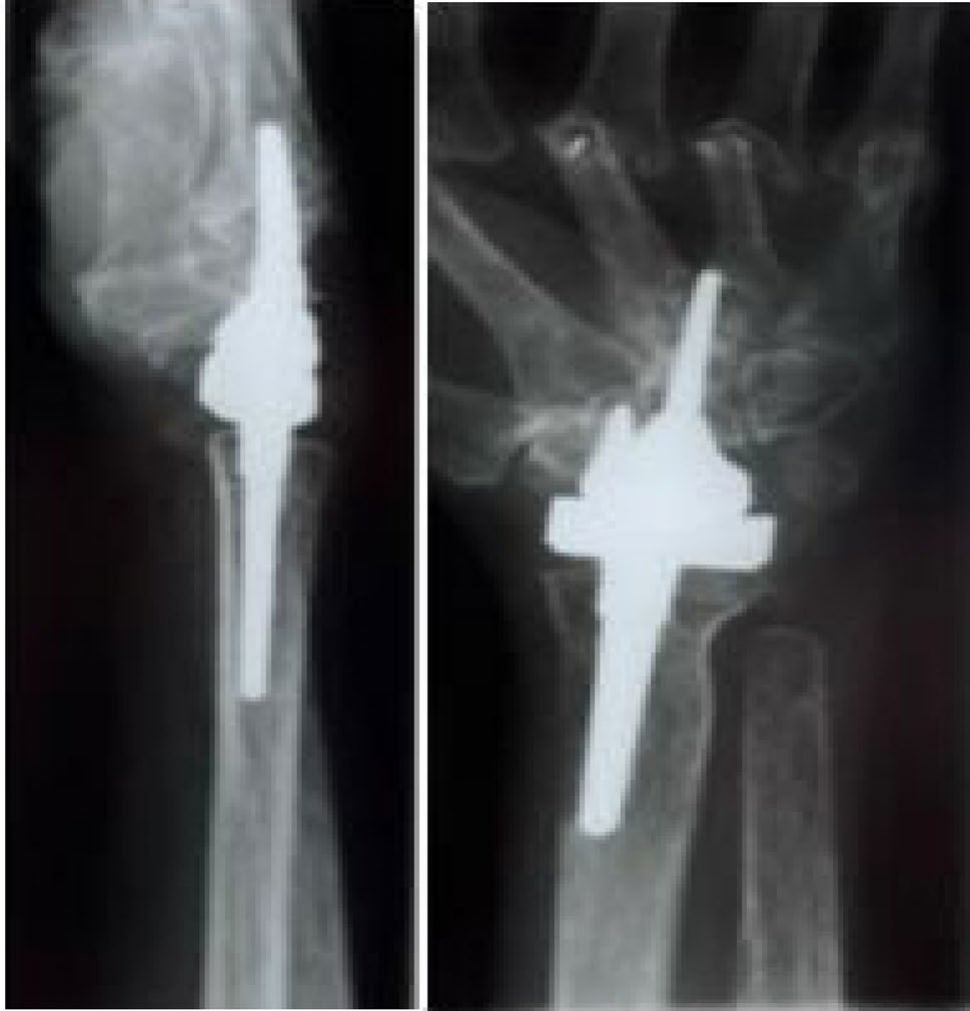
Case 4– Right postoperative X-ray in lateral and a.p. view

Figure 10 shows the situation after 19.1 years after implantation of BIAX-prosthesis:

The postoperative X-rays of the right wrist (lateral and anteroposterior views) demonstrate a total wrist arthroplasty in situ. The prosthesis components are well-aligned, with the radial stem securely anchored within the medullary canal of the distal radius. The carpal component is positioned centrally within the carpal region, with no evidence of dislocation or gross loosening. There are no visible periprosthetic fractures or significant osteolytic lesions. The bone-prosthesis interfaces appear intact, and there is no apparent hardware failure. The surrounding bone shows mild osteopenic changes, but overall, the prosthesis position is satisfactory.

The prosthesis is axially upright in the lateral view, there were no postoperative interventions. The patient does not report any pain at rest or under load. During movement, she achieved 80°/60° in pronation/supination, 30°/50° in extension/flexion and 20°/35° in radial/ulnar abduction. Clinically, the patient showed no swelling, no inflammation and no pain.

## Discussion

In cases of advanced wrist destruction, surgical intervention remains a key option—alongside pharmacologic therapy, physiotherapy, and assistive devices—for preserving joint mobility and alleviating pain. Surgical approaches are employed both preventively and therapeutically, with total wrist arthroplasty (TWA) offering a means of symptom relief while maintaining range of motion in severely degenerated joints [1].

Since the 1960 s, numerous wrist prosthesis designs have been developed, aiming to optimize freedom of movement without compromising stability. Despite significant improvements in surgical technique and implant design over recent decades, wrist arthroplasty still demonstrates relatively high complication rates and poorer outcomes compared to large joint replacements such as hip or knee prostheses [1]. While short-term outcome studies report favorable results, long-term data—particularly beyond 10 years—remain limited. Still some effects such as metal-on-metal disease [2] in wrist arthroplasty are under current investigation [3].

In the present study, the BIAX prosthesis demonstrated a higher 10-year survival rate (70%) compared to the Universal II prosthesis (62%). Literature comparisons for the BIAX prosthesis are sparse; however, Krukhaug et al. [4] reported a survival rate of 78% at 10 years. The survival data for the Universal II prosthesis align with previous reports, which range broadly from 35 to 81% after 10–11 years [5–7]. Long-term follow-up revealed a 15-year survival rate of 60% for the BIAX prosthesis, remaining constant up to 22 years. In contrast, the Universal II prosthesis showed a decrease to 41% at 15 years. Comparative analysis beyond this period is limited, as most existing studies do not extend beyond 10–15 years.

Functionally, the DASH score improved by 12.5% (final score: 33.8%) for the BIAX group after a mean service life of 13.7 years, and by 11.4% (final score: 50.3%) for the Universal II group after 10.4 years. While both scores show improvement, they fall below values reported in previous studies [8]. A low final score was also achieved with the Universal II prosthesis compared to the other studies [7, 9, 10].

Both prostheses achieved the range of motion considered physiologically necessary, as defined by Palmer et al. (1985) for a physiological range of motion was achieved with both prostheses.

The BIAX prosthesis demonstrated superior range of motion compared to earlier studies [11–13], while values for the Universal II prosthesis were consistent with those in the literature [6, 14].

The visual analogue pain scale shows a difference especially under load from a service life of ≥ 15 years. While the BIAX prosthesis shows values of 1 (without load) and 1 (with load), the Universal II prosthesis shows values of 0 (without load) and 3 (with load). This means that the values of the BIAX prosthesis are below the values of comparable studies in which a resting pain of 3 or a range of 0–6 is given [4, 11]. The Universal II prosthesis shows a similar pain level to Gil et al. [6], but a lower resting pain compared to the literature, where values of 0.8 to ≥ 5 were shown [7, 9, 10].

In terms of patient satisfaction, 86% of BIAX patients reported being “very satisfied” or “satisfied,” compared to 78% for the Universal II group—an 8% advantage for BIAX. However, the only “not at all satisfied” rating occurred in the BIAX group due to recurrent postoperative inflammation, noted in a patient with the longest implant survival of 21.4 years. Despite this, the patient exhibited excellent clinical results with no pain, stable radiographic findings, and only limited extension and radial/ulnar deviation (0°, 5°/5°).

Dissatisfaction across both groups was generally due to reduced strength (particularly in supination) and aesthetic concerns such as swelling. Interestingly, the patient with the highest pain scores at follow-up (VAS 4 at rest; 6.5 under load) expressed satisfaction and would recommend the procedure. Conversely, all dissatisfied patients were pain-free but exhibited limited mobility, indicating that pain control alone was insufficient to meet patient expectations. This contrasts with earlier findings [13], where reduced pain—even with limited motion—was a key determinant of patient satisfaction. In our cohort, the lower preoperative pain levels may have led to higher postoperative expectations, reflecting a shift in patient-perceived surgical success criteria [15].

A common finding was the progressive dorsal tilting of the distal prosthetic component or the hand itself, resulting in a limitation of palmar flexion (Figs. 5 and 7). In advanced cases of rheumatoid involvement of the wrist, subluxation or even dislocation of the proximal carpal row is frequently observed; in the carpus, the proximal row is often ankylosed in this malposition. When resecting the proximal carpal row together with the proximal pole of the capitate, the resection angle should be adjusted accordingly to allow for primary implantation of the distal prosthesis component in the correct position. This may help to prevent further progression of dorsal tilting of the wrist.

In the early implantation phase, complications such as dislocation and synovial arthritis occurred more frequently. The most common indication for revision was distal component dislocation, consistent with findings by Damert et al. [16]. Over time, our complication management strategies evolved significantly.

As surgical consequences and learning curve Damert et al. [16] emphasized the importance of alignment and ligament stability in total wrist arthroplasty revision. Our experience confirms that distal dislocation primarily resulted from ligament insufficiency and could be addressed with secondary ligamentoplasty.

In line with Damert [17], patient education and careful preoperative planning are now standard procedures in our institution to improve long-term outcomes.

Cash and Talwalkar [18] proposed revision strategies relying on modular component replacement. Our cohort also benefitted from modularity, avoiding complete explantation in several cases.

The work of Cavalcanti Kußmaul et al. [19] highlights the role of early surgical intervention in carpal pathology to prevent secondary deterioration. Similarly, early TWA in selected patients in our study helped to avoid the need for wrist fusion.

Jump et al. [20] provided perspective on the balance between arthroplasty and arthrodesis in the modern era. In our cohort, arthroplasty was preferred for preserving motion in activities of daily living, although arthrodesis remained a fallback in prosthesis failure.

Boeckstyns and Herzberg [21] documented common complications such as synovial inflammation and loosening. These were reduced in our later cohort through refined soft tissue handling and surgical technique.

Naturally, given the relatively small numbers of wrist endopsrosthesic surgery worldwide this study has several limitations. First, due to incomplete preoperative data in the BIAX cohort, direct comparisons with the Universal II group regarding pre-/postoperative ROM and VAS scores were not possible. Second, the overall sample size of 29 prostheses was too small to allow for broadly generalizable conclusions. Third, the absence of a control group further limits the strength of statistical inference. Nonetheless, this study contributes valuable long-term data on wrist arthroplasty, particularly for the underreported BIAX prosthesis.

## Conclusion

Based on our observations, the BIAX prosthesis demonstrates a clear advantage over the Universal II prosthesis in several key outcome parameters 15 years postoperatively. It shows higher long-term survival probability, a lower complication rate, and more favorable results in terms of patient satisfaction, pain levels on the visual analogue scale, and functional outcomes as measured by the QuickDASH score. While both implants achieved comparable results in preserving wrist mobility, the BIAX prosthesis exhibited a slight superiority in range of motion.

Despite these promising findings, the limited sample size and absence of a control group necessitate larger, randomized controlled trials to further validate and refine these results. Nonetheless, our study provides valuable long-term insights and highlights the BIAX prosthesis as a robust and reliable option in total wrist arthroplasty.

## Data Availability

No datasets were generated or analysed during the current study.
